# Detection of diversity of fetal heart function in pregnancy-induced hypertension patients by ultrasonography in Aljouf Region, Saudi Arabia

**DOI:** 10.4314/ahs.v24i4.25

**Published:** 2024-12

**Authors:** Md Sayed Ali Sheikh, A Alduraywish, Muhannad Faleh Alanazi, Ahmed Baker A Alshaikh, Umme Salma

**Affiliations:** 1 Department of Internal Medicine, College of Medicine, Jouf University, Sakaka, Kingdom of Saudi Arabia; 2 Department of Internal Medicine, Division of Radiology, College of Medicine, Jouf University, Sakaka, Saudi Arabia; 3 Department of Obstetrics and Gynecology, College of Medicine, Jouf University, Sakaka, Kingdom of Saudi Arabia

**Keywords:** Ultrasonography, pregnancy induced hypertension, fetal cardiac function

## Abstract

**Background:**

The common pregnancy illness known as pregnancy-induced hypertension syndrome (PIH) has the potential to harm both the mother's and the fetus' organs to varying degrees. An essential tool for assessing fetal heart function is ultrasound.

**Objective:**

The goal of this study is to use ultrasound to measure the variety of fetal heart function in patients with pregnancy-induced hypertension.

**Material and methods:**

This hospital-based retrospective study was carried out between January 2021 and January 2022 at the Obstetric Department of Maternity and Children Hospital (MCH), Sakaka, Aljouf, Saudi Arabia, to assess the changes in fetal cardiac function in patients with pregnancy-induced hypertension compared to healthy pregnant women in general. The research process involved screening 60 pregnant women for pregnancy-induced hypertension (PIH) and 40 healthy pregnant women for the control group. Each of the 100 pregnant women had a single fetus, and no prenatal abnormalities were found. All eligible patients underwent prenatal ultrasounds in order to collect the cardiac function measures needed to assess the variety of fetal heart functions rapidly and effectively.

**Results:**

When compared to healthy pregnant women, the results of the ultrasound show a higher significance of the fetal thickness of the cardiac septum, left and right ventricular end-systolic and end-diastolic perimeter and area. Additionally, our ultrasonography findings demonstrate that fetal ventricular systolic fractions 1 and 2 (VSF1 and VSF2) were more significant in patients with pregnancy-induced hypertension (PIH) compared to healthy pregnant women in general.

**Conclusion:**

The cardiac functions of the fetus are negatively affected by pregnancy-induced hypertension (PIH).

## Introduction

Pregnancy-induced hypertension (PIH) is a recognized pregnancy-relevant complication that plays a significant role in organ damage and, in extreme cases, mortality in both the mother and the fetus. Approximately 5 to 8% of all pregnant women worldwide are said to be affected by PIH, according to a report 1. After 20 weeks of pregnancy, PIH is typically identified since it describes hypertension as well as alternative systemic functions. Although it may disrupt the placenta early in pregnancy, which causes inflammation and increases damage, the pathophysiology of PIH is yet unknown 2. The most significant adverse effects of PIH are intrauterine death, intrauterine growth restriction, maternal morbidity, and mortality^3^. However, nulliparity, diabetes, obesity, a lack of prenatal care, and other risk factors were linked to pregnancy-induced hypertension^3^. However, one of the main risk factors for pregnancy-induced hypertension is maternal advanced age^4^. According to earlier research, PIH compromises maternal and perinatal morbidity and mortality^5^. According to reports, PIH may harm the mother's heart, impair placental circulation, and develop fetal hypoxia, all of which lead to poor fetal cardiac function and development limitation 6,7. In obstetrics, ultrasound is frequently used to detect structural and functional fetal heart abnormalities. It also makes it easier to evaluate how PIH affects fetal cardiac function. According to several studies, PIH induces thickening of the fetal left ventricular perimeter, area, and septum, which results in greater compromise of fetal cardiac function in PIH mothers^8^. Due to the spasm of small systemic arteries brought on by PIH, the fetus receives insufficient intrauterine oxygen and blood flow, which culminates in higher left ventricular afterload and irreversible left ventricular hypertrophy^9^. However, above these changes were impacts on fetal cardiac function in PIH mother. PIH is a significant pregnancy problem linked to preterm birth difficulties, intrauterine growth retardation (IUGR), intrauterine mortality, birth asphyxia, and potentially newborn death^10^. As well, there is a lack of information on the possible issue of prenatal outcomes in PIH women. for individuals with pregnancy-induced hypertension. In order to prevent compromises in fetal and maternal circumstances and to assure anomalies, regular ultrasonography monitoring of fetal heart function is essential. This will also help provide information that will be useful for developing certain treatments in the future.

## Materials and methods

This hospital-based retrospective study was carried out between January 2021 and January 2022 at the Obstetric Department of Maternity and Children Hospital (MCH), Sakaka, Aljouf, Saudi Arabia, to assess the changes in fetal cardiac function in patients with pregnancy-induced hypertension. 100 pregnant women participated in total, and they were split into case and control groups. The research process involved screening 60 pregnant women for pregnancy-induced hypertension (PIH) and 40 healthy pregnant women for the control group. Each of the 100 pregnant women had a single fetus, and no prenatal abnormalities were found. The inclusion criteria for these investigations were a history of the singleton fetus, a diagnosis of pregnancy-induced hypertension, and the fact that all individuals gave birth in the same hospital. However, we did not include any fetal malformations in the case or control groups because our goal was to see whether PIH had any negative impacts on fetal cardiac function that had been normal before. We made sure there were no fetal malformations following the initial prenatal examination and prior to the onset of PIH because all pregnant women were affiliated with this hospital from the beginning of pregnancy.

The exclusion criteria included patient - deficient information, related medical issues like impaired kidney and liver function, patients' histories of congenital heart disease, and fetal congenital heart disease. The Jouf University scientific ethics committee granted ethical approval.

### Calculation of the sample size

According to the calculation using the formula N = deff u2 *P*(1-P) /d2, where deff is the design effect, N is the sample capacity, u is 1.96 when the confidence coefficient is 95%, and P is the probability value, the current study enrolled a total of 100 participants.

During the research period, we counted 164 pregnant women who received antenatal care at the MCH. Since 10 of the 70 pregnant women who developed PIH stopped visiting the same hospital for follow-up, we excluded those 10 women from the group. Out of 94 pregnant women, 30 had abortions, and 24 stopped receiving follow-up care at the same hospital. We then discovered 40 healthy pregnant women and enrolled them in our study.

### The procedure of data collection

The current study is retrospective and based on a hospital, and all of the data were taken directly from those files. In addition to the fetus ultrasound examination, the data gathered included a thorough history of the pregnant mother and the fetus.

### Ultrasound assessment report of the fetus in both case and control groups

The equipment for scanning the fetus in between 26 and 32 gestational weeks by ultrasonography in two dimensions and on gray scales.

### Periodic examination of the participants

Assessment of the fetal head perimeter, length of the femur, fetal biparietal diameter, abdominal circumference, gestational weeks, and estimation of the maternal amniotic fluid index.

### Ultrasound fetal examination data

The liver, spine, gastric vesicles in the right and left ventricles of the heart, the thickness of the diastolic intraventricular septum (IVS), and the left and right end-systolic and end-diastolic perimeters with the area (LVSP, LVSA, and RVSP, respectively) were all assessed using ultrasound in the current study.

### Estimation of ventricular systolic fraction

The current study also obtained data on the estimated ventricular systolic fractions, which include the ventricular systolic fraction 1 (VSF1), which consists of the ventricular end-diastolic and systolic perimeter and ventricular end-diastolic area, and the ventricular end-diastolic fraction 2.

### Statistical analysis

Using the SPSS 21.0 version of the software, the data were examined. An independent sample t-test was used to examine the data, which were reported as (XS). Analyzed data were counted using (n, %) and the chi-square test. At a p-value of <0.05, the variation was declared statistically significant.

## Results

In this study, 100 pregnant women were enrolled; 60 of them were placed in the case group (pregnancy caused by hypertension) and the remaining 40 in the control group (healthy pregnancy). Ages, mean ages, gestational weeks, mean gestational weeks, gravida, and mean gravida were included for the case (PIH) and control groups.

### Demographic data of case and control groups

In the current study, the criteria for PIH and healthy pregnant women included ranges and mean± SD of age from 22 to 35 years (27.65±3.29), gestation 26 to 32 weeks (26.35±2.57), gravity 1-4 times (1.30±0.41), and comparison with control groups aged from 22 to 35 years (27.14± 3.18), gestation 26 to 32 weeks (26.22±2.51), and gravity 1-4 times (1.28±0.45, respectively) (table 1).

**Table 1:** Characteristics of case and control groups in this study

Variables (Range)	Case (n=60) Mean ± SD	Control (n=40) Mean ± SD	*t*	*P* value
**Age (22-35)**	27.65±3.29	27.14±3.18	0.527	0.609
**Gestational weeks (26-32)**	26.35±2.57	26.22±2.51	-0.221	0.837
**Gravida (1-4)**	1.30±0.41	1.28±0.45	0.192	0.852

### Morphological diversity of the fetal heart

According to Table 2, individuals with pregnancy-induced hypertension had fetal ventricular systolic and diastolic perimeters, areas, and thicknesses that were significantly higher than those in healthy, normal pregnancies at a considered p value< 0.05.

**Table 2:** The diversity of fetal heart among case and control groups

Variables	Case (n=60) Mean ± SD	Control (n=40) Mean ± SD	t	P value
**Diastolic intra-ventricular septum (mm)**	5.69 ± 0.82	4.01 ± 0.44	8.538	<0.001
**Left ventricular end-systolic perimeter (mm)**	56.21 ± 7.11	45.39 ± 6.05	4.38	<0.001
**Left ventricular end-diastolic perimeter (mm)**	63.39 ± 8.25	62.37 ± 6.19	5.361	<0.001
**Right ventricular end-diastolic perimeter (mm)**	72.94 ± 7.87	57.34 ± 5.77	8.76	<0.001
**right ventricular end-systolic perimeter (mm)**	66.38 ± 8.68	56.51 ± 6.49	4.449	<0.001
**left ventricular end-diastolic area (cm^2^)**	3.55 ± 0.89	2.34 ± 0.88	5.617	<0.001
**Left ventricular end-systolic area (cm^2^)**	2.46 ± 0.81	1.92 ± 0.72	3.350	0.001
**Right ventricular end-systolic area (cm^2^)**	2.86 ± 0.79	2.15 ± 0.67	4.573	<0.001
**Right ventricular end-diastolic area (cm^2^)**	3.69 ± 0.85	3.19 ± 0.68	3.161	0.003

### Ultrasound assessment of fetal cardiac function

In the current study, ultrasonography evaluation of the fetal cardiac function in individuals with pregnancy-induced hypertension revealed that the LVSF1 was (0.22± 0.10), the LVSF2 was (0.38± 0.15), the RVSF1 was (0.24± 0.03), and the RVFS2 was (0.35± 0.08). While a healthy, normal pregnancy had values of (0.13 ± 0.04), (0.20± 0.09), (0.16 ± 0.10), and (0.21 ± 0.14). As shown in Table 3, the systolic fractions of the right and left ventricles were more significantly different in the case (PIH) group's fetuses than in the control group's healthy pregnant women (p=value <0.05).

**Table 3:** Ultrasound determines the diversity of fetal cardiac function

Factors	Case (n=60) Mean ± SD	Control (n=40) Mean ± SD	*t*	*P* value
**Left ventricular systolic fraction 1**	0.22 ± 0.10	0.12 ± 0.02	8.012	<0.001
**Left ventricular systolic fraction 2**	0.39 ± 0.18	0.19 ± 0.07	7.206	<0.001
**Right ventricular systolic fraction 1**	0.25 ± 0.04	0.17 ± 0.09	4.735	<0.001
**Right ventricular systolic fraction 2**	0.37 ± 0.09	0.20 ± 0.13)	5.582	<0.001

## Discussion

Our study is the only one ever carried out in Saudi Arabia's Aljouf region, as far as we are aware. In Sakaka, there is just one Maternity and Children's Hospital (MCH), and all patients are referred from outside the city as well. Between January 2021 and January 2022, a total of 60 pregnant women with pregnancy-induced hypertension (PIH) were enrolled and compared with 40 pregnant women who were otherwise healthy. In the current investigation, we concentrate on the ultrasonographic examination of fetal cardiac function. As opposed to the control group, our study discovered the variety of fetal heart functions and their significance. In the case of the group compared to the control group, the present study demonstrated greater significance in the fetal ventricular systolic, diastolic area, perimeter, and thickness of the septal wall, respectively, which expresses an incredibly detrimental effect on fetal cardiac function. Additionally, the fetal right cardiac system should be somewhat larger than the fetal lefteart system due to the increased impact on the right fetal cardiac system due to pregnancy-induced hypertension, which was also supported by other studies^11^, ^12^. Contrarily, we discovered that the right and left ventricles of the fetus in the PIH group had significantly higher systolic fractions than the fetus in the control group. As a result, these findings suggest that the fetal cardiac function of PIH patients varies, which can lead to initial changes in blood flow and hypercoagulation, higher viscosity, and constriction of arteries throughout the body of PIH patients, as well as potential. One of the most serious conditions affecting expectant mothers is hypertension^13^, ^14^, which typically causes spasms in the tiny arteries, increased peripheral vascular resistance, and placental malfunction^15^,^16^. Ultrasonography, however, offers the ability to assess embryonic heart function early, around 6 weeks of gestation; in contrast, a decrease or impairment of cardiac activity indicates abnormalities. The greatest method for early evaluation of various cardiac functions during pregnancy-induced hypertension in women, which aids in the prevention and effective management of both the mother and the fetus, is ultrasonography.

## Conclusion

The cardiac functions of the fetus are negatively affected by pregnancy-induced hypertension (PIH). The current study used ultrasound to identify the variety of fetal cardiac activity, and it discovered that VDP, VSP, and IVS were more significant in PIH patients than in the control group. However, compared to control groups, patients with pregnancy-induced hypertension had significantly greater systolic fractions in both ventricles of fetuses. Because of this, early ultrasound detection of fetal heart function may lessen fetal complications.

## Figures and Tables

**Figure 1:**
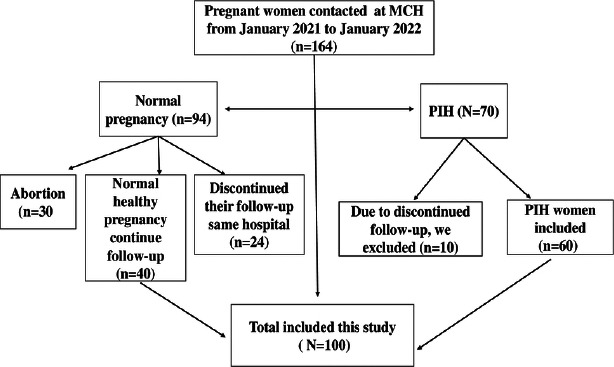
Flowchart for selecting participants at random

## References

[1] World Health Organization (WHO) (2016). WHO recommendations on antenatal care for a positive pregnancy experience.

[2] Stamm ER, Drose JA, Rumack CA, Wilson SR, Charboneau WJ (1998). The fetal heart In Diagnostic Ultrasound.

[3] Rajiah, Mak, Dubinksy, Dighe (2011). Ultrasound of fetal cardiac anomalies. Am. J. Roentgenol.

[4] Tsakiridis S, Giouleka A, Arvanitaki (2021). “Chronic hypertension in pregnancy: synthesis of influential guidelines,”. Journal of Perinatal Medicine.

[5] International Society of Ultrasound in Obstetrics and Gynecology (2006). Cardiac screening examination of the fetus: guidelines for performing the “basic” and “extended basic” cardiac scan. Ultrasound Obstet Gynecol.

[6] Abraham A J M, Bobby Z, Chaturvedula L, Vinayagam V, Syed H, Jacob S E (2019). “Utility of time of onset of hypertension, ADMA and TAS in predicting adverse neonatal outcome in hypertensive disorders of pregnancy”. Fetal and Pediatric Pathology.

[7] Ives C W, Sinkey R, Rajapreyar I, Tita A T, Oparil S (2020). “Preeclampsia—pathophysiology and clinical presentations”. Journal of the American College of Cardiology.

[8] Lv Maoting, Yu Shanshan, Li Yongzhen, Zhang Xiaoting, Zhao Dan (2022). Ultrasound of Fetal Cardiac Function Changes in Pregnancy-Induced Hypertension Syndrome. Evidence-Based Complementary and Alternative Medicine.

[9] Fox R, Kitt J, Leeson P, Aye C Y, Lewandowski A J (2019). “Preeclampsia: risk factors, diagnosis, management, and the cardiovascular impact on the offspring”. Journal of Clinical Medicine.

[10] Ministry of Health and Child Welfare (2015). National Child Survival Strategy for Zimbabwe. 2010-2015.

[11] Al Khalaf S Y, O'Reilly E J, McCarthy F P, Kublickas M, Kublickiene K, Khashan A S (2021). “Pregnancy outcomes in women with chronic kidney disease and chronic hypertension: a National cohort study”. American Journal of Obstetrics and Gynecology.

[12] Park D S J (2021). “Idiopathic intracranial hypertension in pregnancy”. Journal of Obstetrics and Gynaecology Canada.

[13] Zhang Z, Zhang X, Lin X (2019). “Ultrasonic diagnosis of breast nodules using modified faster R-CNN”. Ultrasonic Imaging.

[14] Poniedziałek-Czajkowska E, Mierzynski R, Dłuski D, Leszczynska-Gorzelak B (2021). “Prevention of hypertensive disorders of pregnancy-is there a place for metformin?”. Journal of Clinical Medicine.

[15] Sliwa K, van der Meer P, Petrie M C (2021). “Risk stratification and management of women with cardiomyopathy/heart failure planning pregnancy or presenting during/after pregnancy: a position statement from the heart failure association of the european society of cardiology study group on peripartum cardiomyopathy”. European Journal of Heart Failure.

[16] Easterling T, Mundle S, Bracken H (2019). “Oral antihypertensive regimens (nifedipine retard, labetalol, and methyldopa) for management of severe hypertension in pregnancy: an open-label, randomized controlled trial”. Lancet.

